# Obesity as a public health problem among adult women in rural Tanzania

**DOI:** 10.9745/GHSP-D-13-00082

**Published:** 2013-11-14

**Authors:** Gudrun B Keding, John M Msuya, Brigitte L Maass, Michael B Krawinkel

**Affiliations:** a Justus Liebig University Giessen, Institute of Nutritional Sciences, Department of International Nutrition, Giessen, Germany; b Bioversity International, Nairobi, Kenya; c Sokoine University of Agriculture (SUA), Department of Food Science & Technology, Nutrition and Development Economics, Morogoro, Tanzania; d International Center for Tropical Agriculture, Nairobi, Kenya

## Abstract

**Background:**

For many developing countries, obesity and its sequelae have become a challenge of a magnitude similar to hunger and undernutrition. The main objective of this study was, therefore, to investigate the weight status of women in rural Tanzania with reference to season as well as the link between women's weight, food consumption, and attitudes toward obesity.

**Methods:**

Three cross-sectional surveys in 3 different seasons within 1 year interviewed the same 210 women, ages 17–45 years, from 3 rural districts of northeastern and central Tanzania. These surveys assessed body mass index (BMI), food intake, and dietary diversity through 24-hour recalls, women's attitudes toward obesity, vegetable production, and socioeconomic status.

**Results:**

Although 71% of the women had a normal BMI, 7% were underweight, 16% overweight, and 6% obese. The BMI was correlated with the Dietary Diversity Score (DDS), the Food Variety Score (FVS), with the consumption of foods from the food groups “bread/cakes,” “sugar,” and “tea,” and with the production of exotic vegetables. In a multiple regression model, FVS was directly associated with BMI. When asked to describe the typical characteristics of an obese person, women mentioned more negative than positive characteristics.

**Conclusion:**

The prevalence of overweight and obesity was 3 times higher than that of underweight. Apparently, even in rural areas of Tanzania, a nutrition transition is underway. No direct association was identified between vegetable consumption and BMI. Although this study did not assess behavioral factors, such behavioral factors as activity levels as well as attitudes need to be considered, even in rural settings, to address all facets of malnutrition.

## Abstract (precis)

Even in rural areas of Tanzania, an early stage of the nutrition transition is underway: 3 times as many women were overweight or obese than were undernourished. Overweight and obese women mainly follow a diet characterized by high consumption of bread and cakes (usually fried or baked in oil), sugar, and black tea.

## BACKGROUND

In 1997, a World Health Organization (WHO) consultation formally recognized that the obesity epidemic occurs worldwide and is not limited to the developed world.^1^ Globalization of food markets, urbanization, and economic growth are the main drivers of this development.

In developing countries, profound societal changes and new behavioral patterns have emerged during the last decades that affect nutritional patterns.^2^^,^^3^ An early stage of the nutrition transition—and a typical trend in developing countries—is characterized by increased consumption of cheap vegetable oils that are rapidly integrated into local diets as additional food items. At a later stage, the nutrition transition, as it usually occurs in more wealthy countries, is marked by increased consumption of meat, milk, processed food, and soft drinks together with an increase in the share of food consumed away from home.^4^ At the same time, changes toward a sedentary lifestyle and less physical activity take place. Thus, not only the diet but the whole environment can be “obesogenic”^5^ (promoting excessive weight gain), contributing to increased levels of obesity.

For many developing countries, obesity and its sequelae have become challenges of magnitude similar to those of hunger and undernutrition.^6^^,^^7^ As a result, the coexistence of undernutrition in terms of micronutrients and overnutrition in terms of calories leads to a double burden of malnutrition, not only at the population level^8^ but also in households.^9^^,^^10^ This emerging pattern requires far more attention, and urgently.^11^ Indeed, many poor countries face a “triple burden” of malnutrition—the coexistence of hunger, micronutrient deficiency, and excess intake of calories.^12^

The coexistence of undernutrition in terms of micronutrients and overnutrition in terms of calories leads to a double burden of malnutrition.

Food-based strategies using traditional and locally available agrobiodiversity and promoting consumption of a wide range of foods across nutritionally distinct food groups would benefit not only individuals but also the household and even the community that might be suffering from this triple burden of malnutrition. However, these approaches are still largely neglected and under-researched.

In Tanzania, data on obesity levels exist mainly for urban areas—for example, for Dar es Salaam, where in 1 municipal district, the overall prevalence of obesity was 19.2%, as measured among 1,249 adult male and female subjects^13^; and for Morogoro in central Tanzania, where a prevalence of overweight and obesity was 25% among 100 adults and 40 pupils.^14^ The prevalence of overweight and obesity has been found to be significantly higher in urban Dar es Salaam than in rural Handeni and Monduli for both men and women.^15^ Thus, while the obesity problem has been assessed and is acknowledged in urban areas, trends in rural areas are less investigated.

The present study aimed at investigating the weight status of adult women in rural Tanzania as measured by body mass index (BMI) as well as linkages between weight status, food consumption, and vegetable production. The focus on vegetable production is meant to investigate relationships between agricultural patterns and health.^16^ In addition, we studied women's attitudes toward corpulent (overweight) people, as the social perception of body shape and size can be decisive to behavior, and in many African societies overweight has been associated with wealth, health, and beauty, or in general has a positive connotation.^17^^–^^20^

## METHODS

### Timing and Study Location

For this cross-sectional study, 3 surveys during different seasons within 1 year were conducted, namely, during the dry season in June/July, the short rainy season in November/December, and the end of the dry/beginning of the long rainy season in March/April. The districts and villages for this study were some of those already visited in preceding survey research^21^^,^^22^—6 villages each in 3 different districts of northeastern and central Tanzania: Kongwa, Muheza, and Singida. We chose districts and villages so as to have significant differences in a variety of factors, such as climate, altitude, ethnic group, and distance to urban centers.

### Study Participants

Participants were women selected through systematic sampling in each of the 18 villages by the responsible village extension officer and on the basis of household lists, which were organized by family name (every *k*^th^ household, whereby *k*  =  number of households/sample size). Selection criteria included age between 15 and 45 years and cultivation of vegetables.

Initially, 360 participants were included (120 per district, 20 per village). This number was determined using empirical values of preceding studies and calculating what was possible for a repeated study in the given time and budget frame. Excluded from the analysis were women in the second or third trimester of pregnancy (35 women), as their BMIs cannot be compared with those of non-pregnant women. Similarly, women in the second or third trimester of pregnancy during the second or third survey of this study were then also excluded from the analysis (although they still were allowed to participate). Also excluded were women who had tuberculosis (4 women), according to their own statements, or HIV/AIDS (5 women), according to 7 standard questions on clinical criteria defined by WHO that were asked during the interview, as these conditions also might influence the BMI. Further, 106 women were dropped from the analysis as they were not able to participate in all 3 surveys for various reasons, such as traveling, illness, or who had moved. All remaining women—210 who attended all 3 study sessions—were included, meaning that the same 210 women were interviewed and measured 3 times.

Ethical clearance for this study came from the ethical clearance committee of the faculty of medicine at Justus Liebig University of Giessen, Germany. The study also was approved, and permission for the research was given, by Sokoine University of Agriculture, Morogoro, Tanzania. Oral informed consent was obtained during recruitment from each woman enrolled, as is common practice in communities where some residents are illiterate.^23^

### Data Collection

Women's heights were measured to the nearest 0.1 cm with a person-check (Kirchner & Wilhelm, Asperg, Germany) fixed to a portable wooden measuring board with a foot rest, on which women stood barefoot and without headgear. Weights were measured with a calibrated person standing scale (Seca 862, Seca Co., Hamburg, Germany), on which women were examined barefoot and with minimal clothing according to the FANTA (Food and Nutrition Technical Assistance) protocol.^24^ From body height and weight, we calculated the BMI for each participant (BMI  =  weight in kg/height in m^2^). These anthropometric measurements were taken during all 3 surveys.[Fig f05]

**Figure f05:**
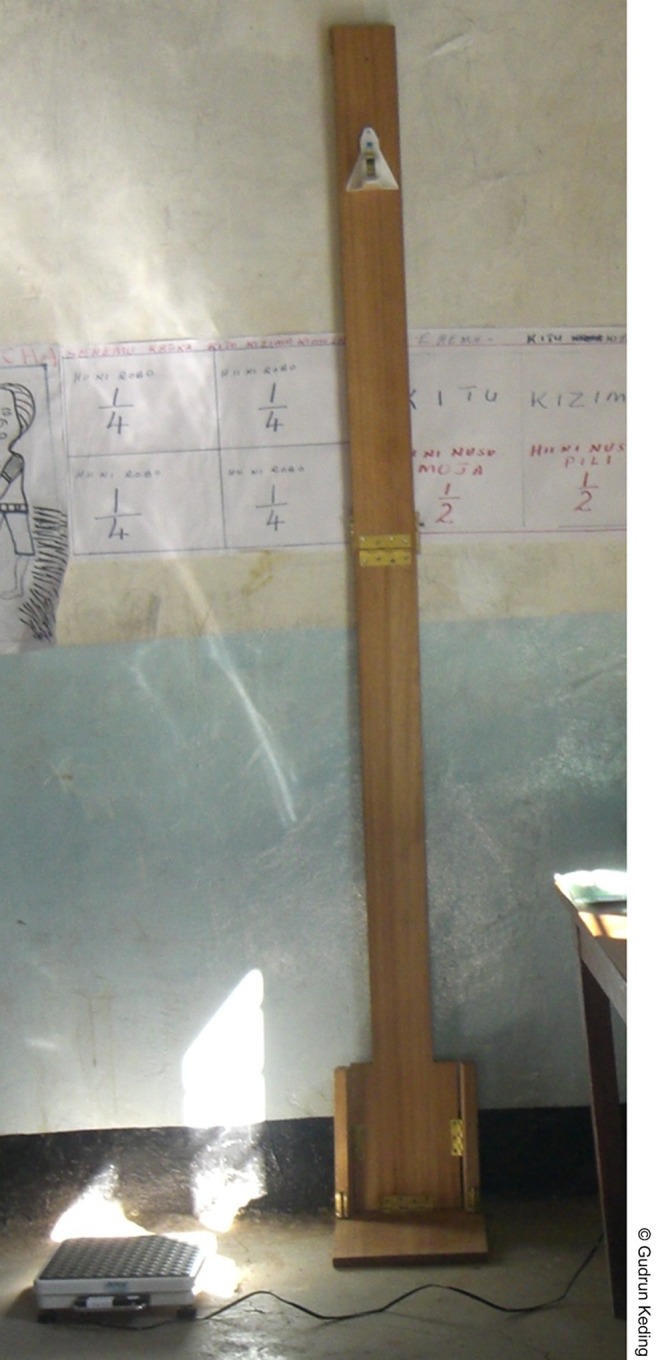
A portable scale and measuring board allowed the researchers to collect anthropometric measurements from study participants during all 3 household surveys.

Participants were interviewed individually during all 3 surveys. The interview, which took 15 to 30 minutes, included a semi-quantitative 24-hour recall of food consumption. To assess the quantity of food eaten, respondents could indicate any 1 of 3 containers to estimate portion sizes; other measures were local cups and spoons. For each food and dish named during this survey, the amount in grams that fits in each of the 3 containers was obtained to serve as a reference for calculating the amounts of foods that participants ate.

Additional questions addressed socioeconomic status (only during the first survey). including household size, distance from village to town, religion, occupation, marital status, and wealth as well as ethnic group and education of participants. The wealth status of each woman was calculated according to the number of possessions including livestock, setting of the house, type of occupation, and whether she sold vegetables.

Regarding vegetable production and collection from the wild, indigenous and exotic varieties were assessed in all 3 surveys in terms of the type and number of types cultivated or collected per woman. Indigenous vegetables included, especially, green leafy vegetables such as amaranth, African nightshade, and spiderplant, but also some fruit vegetables, such as African eggplant, indigenous to Tanzania and East Africa.^21^ Exotic vegetables comprised vegetables introduced from other regions, usually bred for a long time already, such as tomato, onion, cabbage, or carrot.

Finally, during the second survey, the women were asked to mention typical associations with obesity (“a person being very corpulent”) as an open-ended question. Participants' answers were summarized in categories afterwards. Physical activity was assessed during the second and third surveys on a visual analogue scale. Participants rated their own physical activity on an average day on a scale between 0, meaning no physical activity (sleeping), and 10, meaning extremely strenuous physical activity. These subjective data can mainly show changes among individuals between 2 points in time^25^; thus, they were not used in the analysis for this paper.

The questionnaire was developed in English, translated into Kiswahili, in which the interviews were conducted, and translated back into English to cross-check that the correct meaning was maintained. The survey was pretested with 8 women in Arumeru district, Tanzania.

### Data Analysis

All data were checked for normal distribution in order to know whether nonparametric tests (for abnormally distributed data) had to be applied. Women were grouped into 4 categories by BMI status according to WHO categories (2008)^3^ for both women and men ages 15 years or older, namely, underweight (BMI < 18.5 kg/m^2^), normal weight (BMI  =  18.5–24.9 kg/m^2^), overweight (BMI  =  25.0–29.9 kg/m^2^), and obese (BMI ≥ 30.0 kg/m^2^).

Food intake was analyzed for nutrient composition with NutriSurvey for Windows^©^. The Dietary Diversity Score (DDS) was calculated by summing up the number of food groups consumed by an individual over a 24-hour recall period, while for the Food Variety Score (FVS), single foods were counted.^26^ As there is no international agreement on using certain food groups for standardized nutritional analysis,^28^ we allocated 76 different foods to 14 groups.^27^^,^^28^ Then, this system was adapted to food items identified during the survey and used for calculating DDS and FVS.^27^^,^^28^

To characterize the dietary patterns of the participating women, we took an exploratory approach, namely, principal component analysis (PCA). After initially performing the PCA with different numbers of food groups, we found that 12 food groups were best suited to determining dietary patterns among the study participants; all animal sources were grouped together, excluding fish, which is seldom eaten. Five dietary patterns were derived through PCA^29^ based on the mean intake in g/day of the different food groups (mean value from 3 24-hour recalls). For details on the creation of the dietary patterns and criteria for arriving at 5 patterns, see Keding et al.^30^

**Figure:**
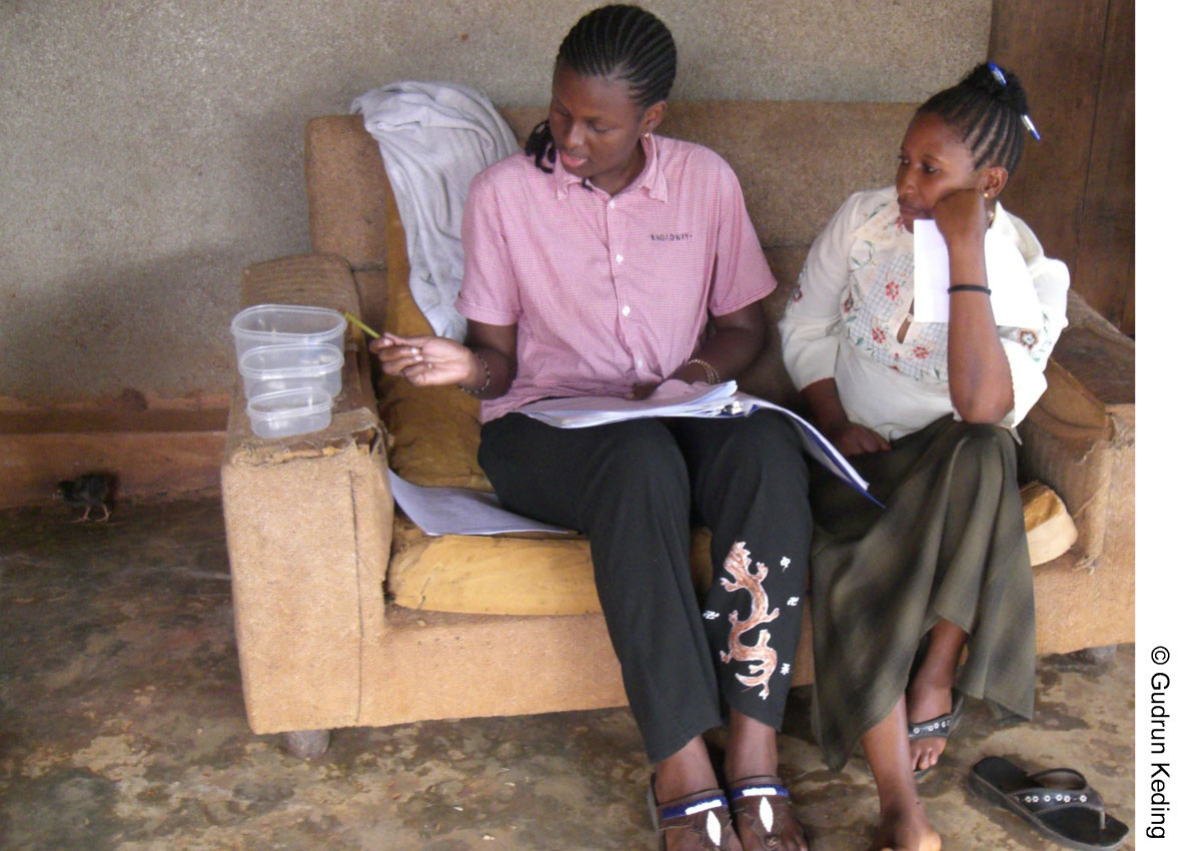
Interviewers showed study participants 3 different-sized containers to help them estimate portion sizes of the foods they ate.

As the BMI data were not normally distributed either for all districts together or for each district individually, we used nonparametric tests to check for differences between seasons (Friedman test) and between districts (Kruskal–Wallis test). As differences were only slightly significant among the seasons (*P* = .045) and not significant among the districts, we used the median BMI of the 3 seasons for all 3 districts combined for further calculations. We used the median BMI instead of the mean, as the data were not normally distributed.^31^

Associations with other variables were tested through both bivariate correlations and multiple regression models. Relationships between categorical variables were assessed with the chi-square (χ^2^) test; and those between continuous variables were tested with the nonparametric Spearman rank correlation coefficient, rho (*ρ*). All statistical analyses were carried out with the Statistical Package for the Social Sciences (SPSS), version 16.0.

## RESULTS

Table 1 shows the main characteristics of study participants. The mean age of respondents was 33.7 years. While in Kongwa and Singida districts, 1 or 2 ethnic groups dominated, in Muheza district several different ethnic groups were present. Most of the participants (90%) had attended primary school, and nearly all of them cultivated or collected indigenous vegetables (98.1%), while only 31.1% cultivated exotic vegetables. The median DDS was 6 different food groups consumed per day, while the mean FVS was 8.4 different foods consumed per day.

**Table 1.** Characteristics of the Women Interviewed in 3 Districts of Tanzania

**Characteristics**	**All Districts**	**District 1 (Kongwa)**	**District 2 (Muheza)**	**District 3 (Singida)**
N	210	52	69	89
Age, mean, y	33.7	30.8	34.6	34.8
Ethnic group (%)				
Bondei	7.6	0.0	23.2	0.0
Gogo	13.3	53.8	0.0	0.0
Kaguru	7.1	28.8	0.0	0.0
Nyaturu	41.9	3.8	0.0	96.6
Shambaa	11.4	13.5	34.8	0.0
Other	18.6	0.0	42.0	3.4
Education (%)				
Illiterate	7.6	7.7	8.7	6.7
Primary school	90.0	88.5	88.4	92.1
More than primary	2.4	3.8	2.9	1.1
Wealth status^a^ (%)				
Low	26.7	30.8	33.3	19.1
Medium	29.0	36.5	29.0	24.7
High	44.3	32.7	37.7	56.2
DDS (median across seasons)^b^	6	5	8	4
FVS (mean across seasons)^c^	8.4	7.2	10.9	7.2
Cultivating/collecting indigenous vegetables (%)	98.1	98.1	99.0	97.4
Cultivating exotic vegetables (%)	31.1	12.2	16.9	53.2

a According to number of possessions, setting of the house, number of livestock, type of occupation, and whether vegetables were sold.

b DDS, Dietary Diversity Score, calculated by summing the number of food groups consumed by an individual over a 24-hour recall period.

c FVS, Food Variety Score, counting single foods over a 24-hour recall period.

Diets with low diversity scores were characterized by a simple but not necessarily unhealthy diet consisting mainly of cereals, vegetables, and pulses, also called grain legumes. With increasing scores, foods such as sugar, beverages (black tea), or animal products were consumed as well. Mean nutrient intakes were calculated using the average of 3 days: the mean energy intake of all participating women was determined to be 1,893 kcal/day, mean protein intake was 60.4 g/day, mean fat intake was 41.3 g/day, and mean carbohydrate intake was 330.3 g/day.

The overall median BMI was 21.7 kg/m^2^ (range 14.9–37.7 kg/m^2^). BMI was highest for the coastal district Muheza and for the November/December (short rains) season (Table 2). In terms of BMI categories, only 7% of all participants were underweight (in Kongwa less than 2%), while 16% were overweight and 6% obese. If the latter 2 categories are combined, more than 20% of participating women had a BMI above 25 kg/m^2^. Detailed data on DDS and FVS have been published elsewhere.^32^

**Table 2.** Median Body Mass Index (BMI) Values and Percent Distribution in 4 BMI Categories of Interviewed Women, by District and Season, Rural Tanzania

				**Distribution by weight category (%)**			
	**N**	**Median (kg/m^2^)**	**Range (kg/m^2^)**	**Underweight (<18.5 kg/m^2^)**	**Normal (18.5–24.9 kg/m^2^)**	**Overweight (25.0–29.9 kg/m^2^)**	**Obese (≥30 kg/m^2^)**
All districts/seasons	210	21.7	14.9–37.7	7.1	71.0	15.7	6.2
Kongwa	52	21.6	17.7–34.7	1.9	75.0	19.2	3.8
Muheza	69	22.5	14.9–37.7	8.7	66.7	14.5	10.1
Singida	89	21.4	16.4–35.2	9.0	71.9	14.6	4.5
June/July (dry season)	210	21.7	14.3–37.3	6.7	71.9	15.2	6.2
November/December (short rains)	210	21.9	15.3–37.7	8.1	70.0	15.7	6.2
March/April (long rains)	210	21.7	12.3–37.2	10.0	68.6	14.8	6.7

### Factors Related to BMI

**Figure 1.**
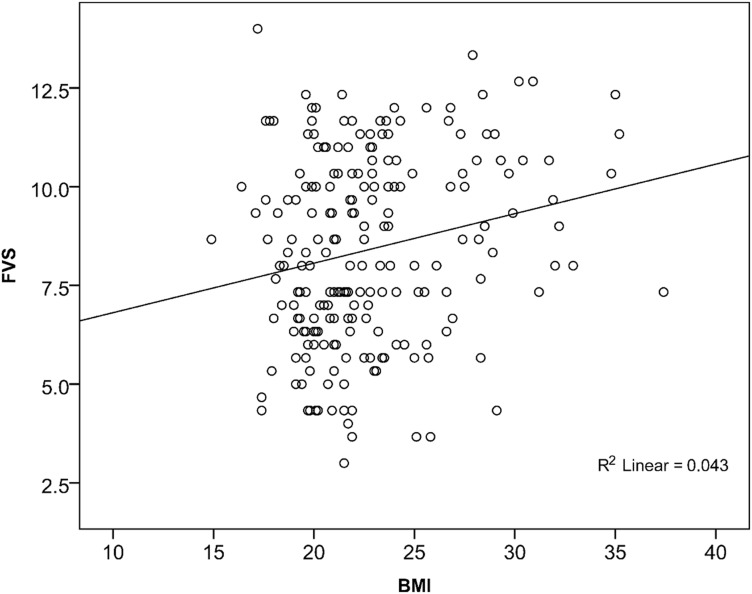
Association Between Body Mass Index (BMI) and Food Variety Score (FVS) (N = 210; *ρ* = 0.204; *P* = .003)

**Figure 2.**
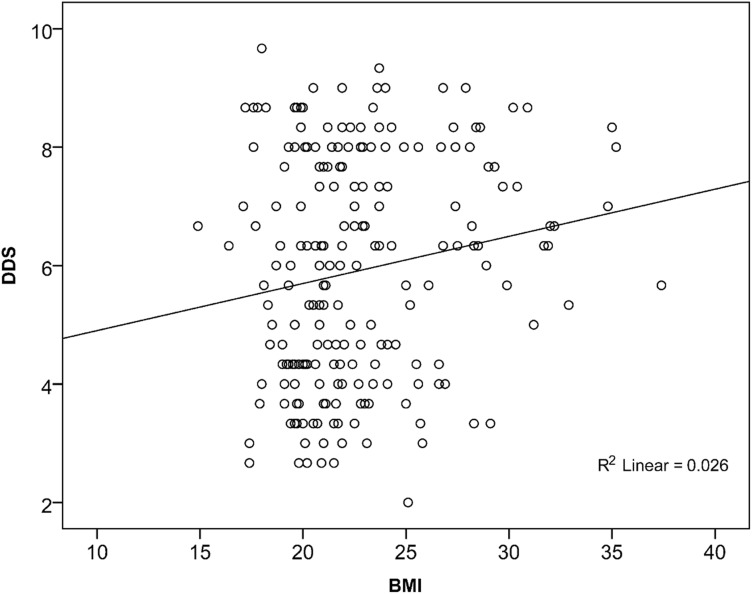
Association Between Body Mass Index (BMI) and Dietary Diversity Score (DDS) (N = 210; *ρ* = 0.147; *P* = .03)

BMI was directly correlated with both the FVS (Figure 1) and the DDS (Figure 2), suggesting that the greater the diversity of foods and food groups eaten, the higher the BMI. Furthermore, BMI was directly correlated with the intake of certain food groups among the 12 food groups used to categorize dietary patterns. These groups were “bread/cakes” (*ρ* = 0.240; *P*<.001), “sugar” (*ρ* = 0.259; *P*<.001), and “tea” (*ρ* = 0.216; *P* = .002). Also, BMI was correlated with the second dietary pattern (*ρ* = 0.192; *P* = .005). Pattern 2 is defined by a high consumption of bread/cakes (usually fried or baked in oil), sugar, and tea. Overweight women had overall higher factor scores for this pattern, and the mean factor score for obese women was highest, meaning that they followed this pattern to a great extent.

BMI was directly correlated with intake of bread/cakes, sugar, and tea.

Bivariate correlation was not found between BMI and any socioeconomic parameter or between BMI and the intake of single nutrients such as fat, protein, vitamin A, or iron, as calculated from the 24-hour recall data. When BMI values were compared with vegetable production/collection, the only significant association found was between production of exotic vegetables and BMI (*ρ* = −0.164; *P* = .017).

We performed multiple regression analyses to study the influence of multiple independent variables on the BMI. Predictors considered were age, dietary pattern 2, FVS, education, and residence (district). The residence of participants and education as nominal variables were transformed into dummy variables to include them in the model and control for them. The significance of this regression model was *P* = .005, while R^2^ was 0.096 and the adjusted R^2^ was 0.064. In this model, only the FVS showed a significant correlation with the BMI (*P* = .046; e^B^ = 1.014) while controlling for the other variables (Table 3). This result suggests that, as the FVS increased by 1 food group, the mean BMI increased by 1.4%; for example, if the FVS increased by 5, the mean BMI increased by 7%.

**Table 3.** Results of Multiple Regression Analysis With ln(BMI) as Dependent Variable, 210 Women from 3 Districts of Tanzania, Mean Across 3 Seasons if Applicable

	**Unstandardized coefficients**		**Standardized coefficients**			
	**B**	**SE**	***β***	**t**	***P*-value**	**e^B^**
(Constant)	2.898	0.079		36.486	.000	
Age	0.003	0.002	0.121	1.748	.08	1.003
Low education	0.068	0.041	0.112	1.642	.10	1.070
High education	−0.092	0.072	−0.086	−1.277	.20	0.912
Kongwa	0.020	0.029	0.053	0.692	.49	1.020
Muheza	−0.032	0.034	−0.093	−0.935	.35	0.969
Dietary Pattern 2	0.022	0.013	0.139	1.682	.09	1.022
FVS	0.014	0.007	0.204	2.008	.046	1.014

Abbreviations: BMI, body mass index; FVS, Food Variety Score; ln, natural logarithm of; SE, standard error.

e^B^  =  inverse of the natural logarithm of B.

### Attitudes Toward Overweight

The open-ended question, “Which typical positive and/or negative properties or qualities would you associate with a person being very corpulent?” sought to elicit participants' attitudes toward overweight and obesity. The number of positive and negative features for a corpulent person named by participants did not show a normal distribution. In general, participants mentioned far fewer positive characteristics (median 0, range 0–6) than negative characteristics (median 3, range 0–7). More than 60% of women (in Muheza, only 47%) gave no example of a positive characteristic of a corpulent person, while nearly all participants expressed 1 or more negative attitudes. When numbers of positive and negative examples were compared, 71% of participants named more negative than positive characteristics for a corpulent person, only 5% named more positive than negative, while 24% were indeterminate. Only one-third of study participants linked overweight with beauty, and even fewer associated overweight with good health.

**Figure 3.**
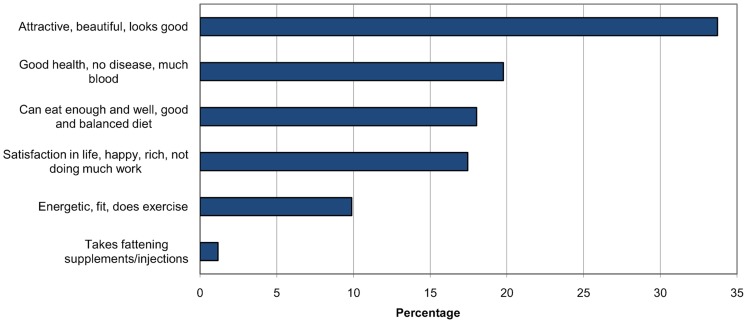
Proportion of Women From 3 Districts in Tanzania Associating Positive Characteristics With a Corpulent Person (multiple answers possible)

**Figure 4.**
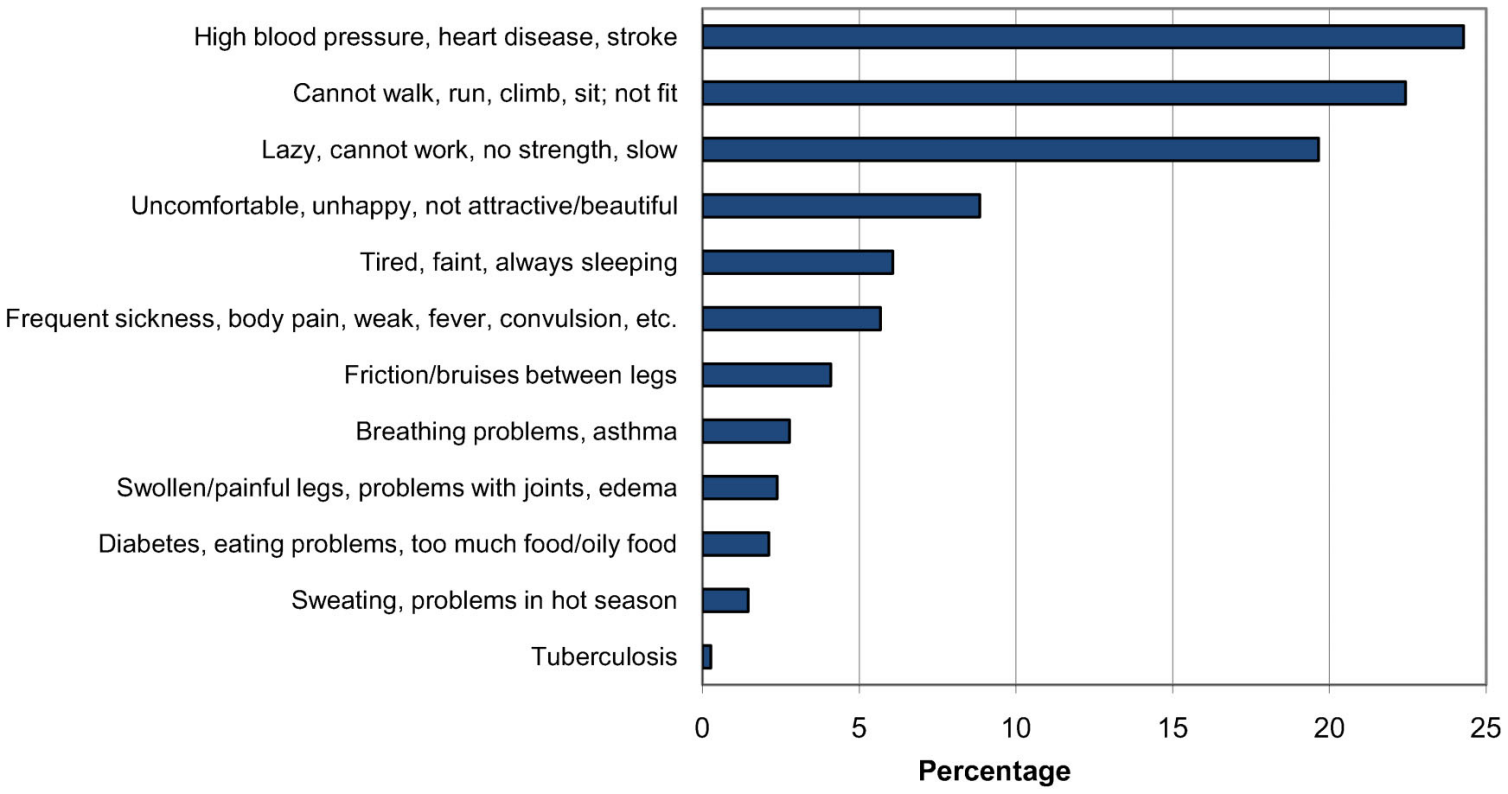
Proportion of Women From 3 Districts in Tanzania Associating Negative Characteristics With a Corpulent Person (multiple answers possible)

The most common positive characteristics were “person is attractive, beautiful, looks good” (34%) and “person has good health, no disease, much blood” (20%) (Figure 3). The most often named negative characteristics were “person has/can get high blood pressure, heart disease, stroke” (24%) and “person cannot walk, run, climb, sit, and is not fit” (22%) (Figure 4). When we grouped the number of negative characteristics named by participants into 3 categories (0–2 negative characteristics named [low], 3 [medium], 4–7 [high]), the number of negative characteristics was found to be significantly associated with the ethnic group of participants (*P* = .01). Respondents' attitudes toward obesity showed no association with their own BMI for any of various measures—negative characteristics only, both negative and positive characteristics, attitude categories, and BMI categories.

## DISCUSSION

In this study on linkages between rural women's BMI, food consumption, attitude toward obesity, and vegetable production as well as on the weight status of rural women in general, the median BMI (21.7 kg/m^2^) of the 210 participants was well within the range of normal weight. However, taking overweight and obesity together, the share of participants with a median BMI above 25 kg/m^2^ was 3 times higher than that of participants with a median BMI below 18.5 kg/m^2^ (indicating undernourishment).

Three times as many women were overweight or obese than were undernourished.

### Overweight/Obesity Prevalence in Rural Versus Urban Settings

In general, data on overweight and obesity in sub-Saharan Africa are scarce. In Tanzania, most studies deal with obesity in urban areas or compare rural and urban areas. For example, in Moshi, a town in the Kilimanjaro region, 70% of 50 surveyed patients with diabetes were found to be overweight.^33^ In Morogoro town in central Tanzania, 100 adults (ages 19–50 years) and 40 pupils (ages 14–18 years) from 4 educational institutions were examined; 25% were overweight or obese. The prevalence of obesity increased with age, and employed persons had higher rates than pupils.^14^ In our study, the prevalence of overweight and obesity was nearly the same (22%) as in the Morogoro study; however, all participants were from rural areas, showing that overweight and obesity are no longer an urban issue only.

A cross-sectional epidemiological study of 545 men and women ages 46–58 years found that the prevalence of overweight and obesity were significantly higher in urban Dar es Salaam than in rural Handeni and Monduli among both men and women.^15^ In a multi-country study with the focus on urban Africa, recent analysis of national BMI data on women found that the prevalence of BMI ≥ 25 kg/m^2^ exceeded that of BMI < 18.5 kg/m^2^ in 17 of 19 countries.^34^ While our study did not compare rural and urban areas, our findings are similar to those of the cross-country study in that the prevalence of overweight/obesity exceeded that of underweight—however, not in urban but rather in rural Africa.

Additionally, the ratio of overweight/obese to underweight participants of 3.1 for the whole study population is similar to the ratio of 3.3 found in a 1996 study of urban Tanzanian women ages 20–49 years, whereas then the ratio was only 1.2 for rural women.^34^ Again, this study confirms a trend of higher prevalence of overweight than underweight, even in rural areas.

Direct comparisons between under- and overweight prevalence often look at the coexistence of obesity and underweight in either mother–child pairs^35^^,^^36^ or in adolescents.^37^ The dual burden of malnutrition in the same household is increasingly reported. However, no clear associations between this dual burden and socioeconomic parameters of households have been found,^35^ and no specific risk factors have been identified so far.^36^

### Association of BMI With Food Intake

BMI values were related to both the DDS and FVS. This relationship indicates that higher dietary diversity and, especially, food variety were associated with higher BMI values (Figure 1 and Figure 2). Multiple regression analysis indicated that, with an increase in FVS, the mean BMI increased. This would be, in general, a positive trend. Nevertheless, when food diversity increased in this study, the additional foods were often sugar, beverages (black tea), or animal products.

Along with overall dietary diversity, the types of food and the amount consumed must be considered.^32^ Foods such as vegetables, legumes, and fruits are often culturally less desired, and, especially, in many sub-Saharan African countries, indigenous vegetables are seen as survival food for poor people.^38^ In general, it must be emphasized that, while obesity and related chronic diseases are becoming more and more serious public health problems in developing countries, at the same time, the prevalence of micronutrient malnutrition is likely to remain high.^39^ Both problems should be addressed by promoting a diet that is not only diverse but also balanced and healthy.

When considering the consumption of different food groups (g/day), we found that only some food groups were positively correlated with BMI. Foods in the group “bread/cakes,” comprising either assorted purchased or homemade types of cakes and breads, usually fried or baked in oil, contain a high amount of fat. Therefore, it was not surprising that participants who consumed a large amount of this food group had a higher BMI. These breads and cakes are usually made of wheat or rice, which replace the traditional starch sources millet and cassava. Although, of course, the consumption of wheat and rice does not, in itself, lead to weight gain, this change implies new processing techniques: Frying in oil replaces cooking in water, and products are often processed further with some kind of fat.^40^ Similarly, in China a vegetable-rich diet was, unexpectedly, found to be associated with obesity. The explanation for this was that the vegetables were stir-fried in oil.^41^

Black tea, a very common drink in Tanzania, is drunk mostly with a large amount of sugar in it, and both food groups—“tea” and “sugar”—were consumed to a great extent by study participants with a high BMI. Regarding sugar, this was not surprising, as the amount of calories consumed was, most likely, often in excess of the need. Even slightly excess food energy intake daily leads to higher body weight in the long run. A study of women in the United States, for example, found that higher consumption of sugar-sweetened beverages was associated with a higher degree of weight gain and an increased risk for developing type 2 diabetes.^42^ In general, an increasing intake of sugared soft drinks has been observed in developing countries,^43^ although not in this study. Nevertheless, in Tanzania as elsewhere, carbonated soft drinks with high sugar content are becoming available even in the smallest corner shops in remote villages. Soon, commercial sugared beverages, particularly carbonated soft drinks, may become a key contributor to an epidemic of overweight and obesity in rural Tanzania, as they have elsewhere.^43^^,^^44^

### Association of BMI With Vegetable Production

The production of exotic vegetables (number of types that a woman cultivated) was inversely associated with BMI; that is, women who grew exotic vegetables were likely to have lower BMI values. This association was not very strong, but it is rather puzzling, as the production of exotic vegetables—in contrast to that of indigenous vegetables—is usually associated with knowledge and a certain degree of wealth, because seeds and further inputs have to be purchased. Several studies suggest a positive association between wealth and BMI in developing countries.^3^^,^^6^^,^^45^ Thus, it would be expected that exotic vegetable production would be associated with a higher BMI. However, our study found the opposite. We did not assess it in this study, but it is a relevant question whether exotic vegetables are mainly—or even exclusively—sold and so do not directly affect household food and nutrition security, but rather only contribute to the general wealth of a family. Our study could not confirm an association of wealth with BMI, possibly because all study participants had rather similar wealth status, as is typical in rural areas, and because the association between wealth and BMI might be, in general, more pronounced in urban areas.

### Attitudes Toward Overweight

It was not clear whether study participants associated a corpulent body with wealth, health, and beauty, as studies in Mauritania^17^ and Morocco^19^ have found. In Tanzania, as well, it has been suggested that especially overweight and obese women are perceived as beautiful; they are admired and respected, while skinniness is associated with illnesses, especially HIV/AIDS, and, therefore, is not desirable.^46^ Thus, we expected that study participants would name more positive than negative characteristics of corpulence. However, the share of participants naming more negative than positive characteristics was much greater than those indicating mostly positive features.

Respondents cited more negative than positive characteristics of overweight people.

The circumstances under which interviews were conducted must be considered carefully; participants may have given answers that they expected the interviewer wanted to hear, as may happen in any interview. Nevertheless, as participants mentioned a number of “technical medical terms” associated with corpulence, such as diabetes and edema, they must have previously heard about problems related to overweight and were, apparently, already sensitized to these topics. Assuming that all women said what they thought, the question arises why the majority of these study participants in rural Tanzania no longer thought in the traditional way but instead were already sensitized to obesity as a health problem.

Regarding the correlation between women's attitudes and ethnic group, it is possible that women of different ethnic backgrounds have different attitudes toward body image and overweight. Different body image perceptions have been identified among Australian school children from varying ethnic groups.^47^ Similarly, a cross-cultural study with participants from Europe, India, Japan, Oman, the Philippines, and the United States found cultural differences in the drive for thinness as well as attitudes toward eating.^48^ In our study these different perceptions by ethnic groups coincided with differences among the districts, which are inhabited by different ethnic groups. Consequently, even within the rural area of one country, ethnic differences need to be considered.

In general, the BMI cutoff point of 25 kg/m^2^ must be reconsidered. In the United States, the link between weight and mortality has recently been assessed; underweight, obesity, and, especially, extreme obesity (BMI ≥ 35 kg/m^2^) were associated with increased mortality, yet, overweight (BMI  =  25 to < 30 kg/m^2^) was not.^49^ Thus, while obesity is an obvious health risk, this is not necessarily true for overweight, depending on the age, sex, and ethnicity of a person. Especially in countries or regions with recurrent food shortages and high disease prevalence, it should be considered that people who are slightly overweight may be healthy and have higher chances of longevity than lean people.

## CONCLUSIONS

For this study the same participants were tracked during 3 different seasons in 1 year, thus creating rather robust data regarding seasonal changes in food intake and weight status, although seasonal differences were minor or nonexistent. Furthermore, as most studies in Tanzania so far have focused on urban areas or rural–urban comparisons, this study makes a new contribution by focusing on rural residents only.

In general, data obtained through interviews always must be handled with care, as interviews have some disadvantages that cannot be avoided. Dietary recall, for instance, relies on the respondent's memory as well as on her ability to estimate portion sizes. However, since the survey was interviewer-administered and participants did not have to record their food intake themselves, the data were collected from all respondents in a consistent manner.^50^

In this study of adult women in rural Tanzania, overweight and obesity were more prevalent than underweight. The 3 main reasons for the obesity epidemic in developing countries are changing food consumption habits, cultural attitudes toward overweight/obesity, and decreasing physical activity.^4^^,^^8^ Our study found an indication of only the first reason, namely, changing food consumption habits, when comparing to former eating habits assessed in a previous study;^40^ no indication was found for cultural attitudes and physical activity was not assessed.

The fact that participants had more negative than positive associations with overweight and obesity indicates their awareness of the resulting problems. This may be relevant for public health interventions aiming at prevention at the population level as well as the individual level.

While it can be argued that the high BMI values of people in urban areas of Tanzania are, most likely, due to changing lifestyles, food consumption habits, and physical activity patterns, it still needs to be determined how a similar trend toward high BMI is possible in rural Tanzania. Even without the presence of Western fast food chains, consumption of cheap vegetable oils is increasing. An early stage of the nutrition transition is obviously underway, even in rural areas. No direct association between vegetable consumption and BMI was found. Along with vegetables as part of a balanced diet, behavioral factors, including attitudes and activity levels, need to be considered even in rural settings to address all facets of malnutrition.

An early stage of the nutrition transition is underway in Tanzania, even in rural areas.
